# Computational Identification and Characterization of New microRNAs in Human Platelets Stored in a Blood Bank

**DOI:** 10.3390/biom10081173

**Published:** 2020-08-12

**Authors:** Jersey Heitor da Silva Maués, Caroline de Fátima Aquino Moreira-Nunes, Rommel Mário Rodriguez Burbano

**Affiliations:** 1 Laboratory of Human Cytogenetics, Institute of Biological Sciences, Federal University of Pará, Belém, PA 66075-110, Brazil; rommel@ufpa.br; 2 Laboratory of Molecular Biology, Ophir Loyola Hospital, Belém, PA 66063-240, Brazil; 3 Laboratory of Pharmacogenetics, Drug Research and Development Center (NPDM), Federal University of Ceará, Fortaleza, CE 60430-275, Brazil

**Keywords:** platelet concentrate, microRNA, sRNA-seq, storage lesions, platelet hyperactivity

## Abstract

Platelet concentrate (PC) transfusions are widely used to save the lives of patients who experience acute blood loss. MicroRNAs (miRNAs) comprise a class of molecules with a biological role which is relevant to the understanding of storage lesions in blood banks. We used a new approach to identify miRNAs in normal human platelet sRNA-Seq data from the GSE61856 repository. We identified a comprehensive miRNA expression profile, where we detected 20 of these transcripts potentially expressed in PCs stored for seven days, which had their expression levels analyzed with simulations of computational biology. Our results identified a new collection of miRNAs (miR-486-5p, miR-92a-3p, miR-103a-3p, miR-151a-3p, miR-181a-5p, and miR-221-3p) that showed a sensitivity expression pattern due to biological platelet changes during storage, confirmed by additional quantitative real-time polymerase chain reaction (qPCR) validation on 100 PC units from 500 healthy donors. We also identified that these miRNAs could transfer regulatory information on platelets, such as members of the let-7 family, by regulating the *YOD1* gene, which is a deubiquitinating enzyme highly expressed in platelet hyperactivity. Our results also showed that the target genes of these miRNAs play important roles in signaling pathways, cell cycle, stress response, platelet activation and cancer. In summary, the miRNAs described in this study, have a promising application in transfusion medicine as potential biomarkers to also measure the quality and viability of the PC during storage in blood banks.

## 1. Introduction

Platelet concentrate (PC) transfusions are widely used to save the lives of patients suffering from acute blood loss and are more often used in supportive prophylactic therapy for patients with various hematological diseases [1].

This blood component requires special storage in blood banks, normally being stored up to a maximum of five days at a temperature of 22 ± 2 °C, with gentle and continuous agitation, because even under ideal storage conditions, the PC can undergo modifications or degradations known as platelet storage lesion (PSL), a term introduced by Murphy et al., in 1971 [2], for describing the multifactorial mechanisms of this problem, which include the methods of collection, processing, storage, handling before or after collection, and expiration date [3,4,5]. In Brazil, the validity of the PC is three to seven days, depending on the plasticizer of the conservation bag [6]. More generally, the storage time of the PC depends on local legislation and the additive solution, regarding whether or not to use a pathogen inactivator [7,8].

Longer platelet storage duration has not been recommended due to the possible risk of bacterial contamination. Therefore, research on updating screening of molecular biomarkers to assess and monitor the physiological viability of platelets in PC has gained strength in recent years, mainly to identify PSL [9,10].

The most common changes in platelets during storage in PCs are morphological and physiological changes, platelet activation related to exposure to foreign surfaces, trauma, low pH, platelet agonists such as thrombin and ADP, promoting changes in membrane glycoproteins, proteolysis, and expression of platelet surface receptors, culminating in the release of microparticles (MPs) rich in microRNAs (miRNAs) [5,10,11,12,13,14,15]. Tests of mitochondrial dysfunction show that the overall bioenergetic health of stored platelets is significantly lower as compared with fresh platelets, suggesting that stored platelets are more susceptible to oxidative stress [16,17] and apoptosis [10,18].

MicroRNAs (miRNAs) comprise a class of molecules with a biological role capable of acting together to mediate sequence-specific regulation by repressing or degrading mRNAs at specific non-translated binding sites [19] and are a relevant tool for understanding storage lesions, proven by studies on stored platelets that continue to translate mRNA proteins [20,21,22,23]. Platelets contain an abundance of RNAs, miRNAs and have fully functional mRNA splicing machinery. In this context, platelet miRNAs can regulate the levels of expression of platelet mRNA and, consequently, proteins [24,25,26].

Our previous studies have shown miRNAs to be a promising tool for measuring the quality of PCs stored in blood banks. We found that the inverse expression relationship between miR-127 and miR-320a allowed us to identify PC bags that could still have physiologically normal (non-activated) platelets [20]. In addition, we identified another 14 miRNAs expressed differentially, comparing a control PC from the first day of storage with the PCs on each of the subsequent five days of storage from day one to day seven [21]. In this study, we used a new approach with bioinformatics and computational biology methods to identify and characterize the profile of new miRNAs and their sequence variants in platelet concentrates stored for seven days in a blood bank.

## 2. Methods

### 2.1. Dataset Analysis

In this study, we used the data available in the Gene Expression Omnibus (GEO) database with accession number GSE61856, studied by Pontes et al. [20]. The data were generated with small RNA-sequencing (sRNA-Seq) from PCs using an Illumina HiSeq 2000 platform. In this experiment, sixteen PC bags tubes were used, then, cut into six equal parts and maintained for seven days of storage. PC pooled miRNAs, sufficient to perform the sequencing, were extracted on each day of the seven days. The first day was the high-quality control of platelets and the seventh day was the low-quality control of platelets [20].

Human platelets were collected from healthy donors according to international standard protocols and stored at 22 ± 2 °C for 7 days. The small RNA population was evaluated after the first day (PC-1), the second day (PC-2), the third day (PC-3), the fourth day (PC-4), the fifth day (PC-5), and the seventh day (PC-7), of storage at 22 °C. Thus, small changes in the RNA population over the days were assessed with sRNA-Seq [20].

### 2.2. MicroRNAs Prediction in PCs

The SRA (sequence read archive) from the seven PC samples were downloaded from GSE61856 and used as input in the sRNAbench and sRNAtoolbox [27], latest version, updated in 2019. Reads generated with sRNA-Seq were processed with sRNAbench and showed high quality >90% in all six libraries.

We performed the reading mapping pipeline in genome and library mode, in which both modes used a common preprocessing step, mapping, expression profile, and miRNA prediction [28]. For this, the 3’ adapter sequence was trimmed, and the sequence length distribution was analyzed. Sequences with a reading length between 15 and 27 nucleotides (nt) were aligned with the miRNA precursor sequences (pre-miRNA) of human miRBase, version 22 [29].

To this analysis, we applied the standard sRNAbench parameters as follows: (i) minimum length of adapter that needs to be detected, 10; (ii) alignment type, Bowtie seed alignment (GRCh38_p10_mp), seed length for alignment 20, minimum read count 2, minimum read length 15, allowed number of mismatches 1, the maximum number of multiple mappings 10; (iii) MEAN quality filtering was used, 20; and (iv) the annotation used miRNAs for species hsa (*Homo sapiens*).

### 2.3. MicroRNA Expression and Quality Analysis between Different PCs

We assessed the level of miRNA expression by counting per million reads (CPM) using the following formula: CPM = (reads number of one miRNA)/(total mapped reads to all annotated miRNAs) × 10^6^. We defined a miRNA expressed in a PC with a CPM > 1 in more than 50% of the six PC samples. The PC-specific miRNA was defined as a miRNA expressed exclusively in PC on the first day (PC-1), was used in this study as the platelet quality control.

Because PCs are stored in a blood bank for a maximum period that varies between three and five days, depending on the plasticizer in the conservation bag [6], our analyses considered the first day as the high-quality control of platelets and the seventh day corresponded to the low-quality control of platelets [20].

In our computational results obtained with sRNA-Seq, we analyzed miRNAs expressions according to the methodology described by Pontes et al. [20] to check which PC bags were in good condition for transfusion. This method compared the relative expression between miR-127 and miR-320a [30]. When miR-127 presented a lower expression (<80%) as compared with miR-320a, storage lesions in this blood component were considered, suggesting the blockage of the PC bag for transfusion.

The PC bag was considered to be suitable for transfusion when it had one of the following possibilities: (i) expression of miR-127 > miR-320a, (ii) equal expression between miR-127 and miR-320a, (iii) and a difference less than 20% in expression between these two miRNAs. In this study, the results generated for the expression levels of miRNAs were analyzed using computational biology simulations, employing unsupervised grouping and principal component analysis (PCA).

### 2.4. Validation of microRNAs by qPCR on 100 PC Units

Each PC which was used contained platelets from five healthy donors. Therefore, validation was performed in 500 donors (250 men and 250 women in the age group that comprises young adults, between 18 and 40 years old). All biosafety policy guidelines were applied in the involved laboratories, with the approval of the Ethics Committee (#194, 196, approval 17 October 2012).

Seven days of storage were analyzed, and the first day was used as the control for the following days, totaling five comparisons of expression levels for each of the six miRNAs identified, accounting for 30 analyses. As the validation occurred in 100 PC units, we totaled this validation in 3000 analyses. Mir-191 was selected as an internal control for miRNA input and reverse transcription efficiency because the miRNA was most highly expressed on seven different days of storage [20]. All real-time quantitative PCR (qPCR) reactions were performed in triplicate for both miRNAs. Expression levels of miR-486-5p, miR-92a-3p, miR-103a-3p, miR-151a-3p, miR-181a-5p, and miR-221-3p were isolated from 100 PC units with mirVana^TM^ miRNA Isolation Kit (Thermo Fisher Scientific, Waltham, MA, USA).

The miRNA was reverse transcribed using the TaqMan^®^ MicroRNA Reverse Transcription Kit, according to the manufacturer’s protocol (Thermo Fisher Scientific, Waltham, MA, USA). The qPCR analyses were performed with TaqMan^®^ microRNA assays (Thermo Fisher Scientific, Waltham, MA, USA) for miR-486-5p (ID 001278), miR-92a-3p (ID 000431), miR-103a-3p (ID 000439), miR-151a-3p (ID 002254), miR-181a-5p (ID 000480), miR-221-3p (ID 000524). Then, complementary DNA was amplified by qPCR using the TaqMan Universal Master Mix II with UNG (Thermo Fisher Scientific, Waltham, MA, USA) on a Rotor-Gene Q (Qiagen, Hilden, Germany).

### 2.5. IsomiR Annotation Analysis

In this analysis, we detected the miRNAs sequence variants called isomiR [31]. To detect isomiRs, we applied the following steps for the sRNAbench pipeline: (i) mapping the reads with the pre-microRNA genome or sequence using the Bowtie seed option [32], (ii) determining the coordinates of the mature microRNA, (iii) clustering of all reads that mapped within a window of the canonical sequence of the mature microRNA (miRBase), and (iv) applying a hierarchical classification of the variants [27].

Then, a subsequent analysis was performed to detect multiple NTA sequence variants which involved non-templated additions (enzymatically addition of a nucleotide to the 3′ end, i.e., adenylation and uridylation) that included NTA(A), number of reads with a non-templated A (adenine) addition; NTA(U), number of reads with a non-templated U (uracil) and (T) thymine addition; NTA(C), number of reads with a non-templated C (cytosine) addition; and NTA(G), number of reads with a non-templated G (guanine) addition.

The second class of length variants included 5′ and 3′ trimming and extension in the following forms: lv3pE, number of reads with 3′ length extension (longer than the canonical sequence); lv3pT, number of reads with 3′ length trimming (shorter than the canonical sequence); lv5pE, number of reads with 5’ length extension (longer than the canonical sequence); lv5pT, number of reads with 5′ length extension (shorter than the canonical sequence); and mv, number of reads classified as multiple length variants [28]. We organized the results of this analysis by the ranking of isomiRs identified in PCs and mature miRNA most expressed according to individual reading counts (CPM expression).

### 2.6. MicroRNAs Target Prediction

We investigated the possible target genes of the miRNAs most expressed in PC with the TargetScan algorithms, version 7.2 (http://www.targetscan.org/vert_72/), miRTarBase, version 8.0 (http://mirtarbase.cuhk.edu.cn/php/index.php), and miRDB, version 6.0 (http://www.mirdb.org/statistics.html), to identify possible miRNA–gene interactions. TargetScan is a predictor that generates predicted interactions, while miRTarBase and miRDB provide validated interactions [33,34,35].

For this analysis, we applied an information retrieval feature known as target mining in the miRWalk predictor, version 3.0 (http://mirwalk.umm.uni-heidelberg.de/), which hosts the three aforementioned predictors, to obtain the information from miRNA–gene interactions that were organized in a table with various predictive metrics, which were considered for binding probability (*p* > 0.95) [36], sites preferably conserved within the 3’ UTR (untranslated region) and validated interactions [37].

Three subsets of data were analyzed and listed as follows: (1) TargetScan + miRDB, (2) TargetScan + miRTarBase, and (3) miRDB + miRTarBase. For further investigative analysis, we considered the miRNA–gene interactions that were identified at the intersection of the Venn diagram of these three subsets. For this analysis, we used the Venny tool (https://bioinfogp.cnb.csic.es/tools/venny/).

### 2.7. Construction of the microRNA—Gene Interaction Network

The subset files containing the predicted and validated interactions, identified in the previous analysis, were used in the construction of miRNA–gene interaction networks. For that, we filtered only the predicted and validated interactions of interest to remove those annotated symbol genes for more than one Refseq identifier. Then, we simulated the construction of two miRNA–gene interaction networks for the two predicted and validated interaction files (1) and (2), respectively, which were loaded and viewed in Cytoscape, version 3.8.0 (https://cytoscape.org/).

The two networks were merged according to the tutorial (http://manual.cytoscape.org/en/stable/Merge.html) with an intersection operator to obtain a single miRNA–gene interaction network common to the two previous networks, formed exclusively by interaction data predicted and validated. The central region of the network with the densest connections was detected with CytoHubba [38]. We applied the maximal click centrality (MCC) method to identify miRNA–gene interaction clusters.

### 2.8. Functional Enrichment Analysis

The target genes of the miRNA–gene interaction network from the previous analysis were used for functional enrichment with the tool the Database for Annotation, Visualization and Integrated Discovery (DAVID, version v6.8) [39]. These target genes were organized in a list containing only the Refseq identifier of the species (*Homo sapiens*).

To avoid redundancies in terms, high-stringency classifiers with a similarity threshold and multiple linkage threshold equal to 0.50 were applied. The most significant terms for each gene were obtained from functional annotation clusters GO (Gene Ontology) [40] and from KEGG pathways (Kyoto Encyclopedia of Genes and Genomes) [41] with significant values noted with Log_10_, *p*-value < 0.05.

### 2.9. Statistical Analysis

All statistics were performed using R (https://www.r-project.org). The averages obtained with the non-parametric tests were calculated using the compare_means function. Principal component analysis (PCA) with the FactoMineR package [42] was used for exploratory investigation of multivariate data from miRNA using the prcomp function. The unsupervised grouping and heatmaps were built with the heatmap function.2. The correlation coefficients of the isomiRs were calculated with the following functions: cor and rcorr. Correlations with *p*-value > 0.01 were considered not significant. The ANOVA test was used to test the means among the miRNA variants.

## 3. Results

### 3.1. Data Preprocessing and Abundance of microRNAs in Platelet Concentrate

The results of sRNA-Seq analysis from PCs of the GSE61856 repository, made with sRNAbench, showed that after a preprocessing of the data, more than 95% of the reads were recovered, which mapped between 81 and 85% of unique regions of the genome, with coverage genomics comprised of 95%, (Table 1). We observed that the amount of read counts (RC) detected for miRBase hairpins showed a decrease from the fourth to the fifth day, increasing only on the last day. The same pattern was observed for mature miRNAs that presented more than 35% of miRNA expressed on the last day of storage (PC-7) (Table 1).

**Figure 1:**
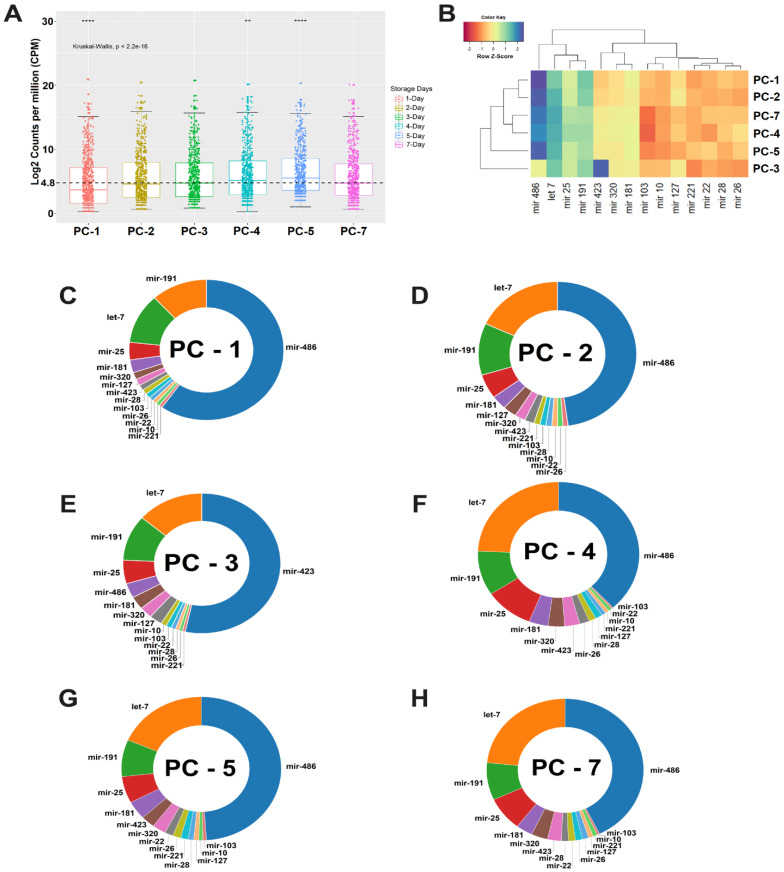
(**A**) Average levels of normalized expression in Log_2_ counting per million reads (CPM) of miRNA in platelet concentrates (PCs). The average expression was 4.8 CPM. Boxplot is designed from the 75th to the 25th percentile. The vertical lines above and below the box define the maximum and minimum values and the dots indicate outliers, the horizontal line inside the box represents the median value. Kruskal–Wallis test (*p*-value <0.001) was applied to compare the means between the six groups (** *p* < 0.01, **** *p* < 0.0001); (**B**) The heatmap shows an expression profile defined by the most abundant miRNA families in all PCs. Z-score was the metric applied to infer the best clustering between miRNA families. Gradients with a red tendency represent families of miRNAs with a lower Z-score and gradients with a blue tendency with a higher Z-score; (**C**–**H**) Donut chart shows the ranking of miRNA family positions on all PCs. PC, platelet concentrate.

The abundance of miRNAs in stored PC bags described in Table 1, shows an increase in the number of reads until the third day of storage (PC-3), followed by a decrease in PCs on the fourth (*p* = 0.023) and the fifth (*p* = 8.9E-08) days, with an increase in the median expression, exceeding the average of normalized expression of 4.8 CPM (Figure 1A). As a direct consequence of two more days of storage, the PC on the seventh day (PC-7) presented the largest number of reads and 939 miRNAs, which represented an increase of 2.5% observed after seven days of blood collection, confirmed by the decrease in the median. These results confirm that storage for more than five days in a blood bank causes a decrease in the levels of miRNA expression in the PC.

Then, we analyzed the miRNA annotation files obtained with sRNAbench. We found a group of very abundant miRNA families that were shown in the unsupervised cluster analysis, represented by mir-486, let-7, mir-25, and mir-191. A second group of families are also grouped, whose members are represented by mir-423, mir-320, mir-181, mir-103, mir-10, mir-127, mir-221, mir-22, mir-28 and mir-26 (Figure 1B).

We provided a panoramic view of the distribution of these miRNA families in all PCs (Figure 1C–H), where we could see the predominance of the mir-486, let-7, mir-25, and mir-191 families, except for on PC-3 which presented the most abundant mir-423. From this information, we also identified the top-20 miRNAs most expressed. Table 2 shows the detailed information on the precursor (pre-microRNA) and the mature miRNAs identified in this study from the annotation files. It shows a decrease in the level of expression of these miRNAs until PC-5 and an increase after two days on PC-7. In Supplementary Materials File S1, we present detailed information on several canonical miRNAs, with specimens that had their functions elucidated in platelets, in addition to a wide variety of miRNAs that have not been described in the literature.

### 3.2. Measurement of microRNA Expression in PC

After analyzing the miRNA expression files generated with sRNAbench, we identified the top-20 miRNAs most expressed in the PC, represented by the following members: miR-486-5p, miR-191-5p, let-7i-5p, miR-92a-3p, miR-181a-5p, let-7a-5p, let-7b-5p, miR-320a-3p, miR-127-3p, miR-103a-3p, miR-26a-5p, miR-151a-3p, miR-423-5p, let-7g-5p, miR-22-3p, miR-221-3p, let-7f-5p, let-7d-5p, miR-28-3p, and miR-423-3p (Table 2). Considering those 20 miRNAs most expressed that were described, we found that they are part of a family of the 14 most abundant miRNAs in platelets, in which some families present more than one mature miRNA, such as let-7 and mir-423.

**Figure 2:**
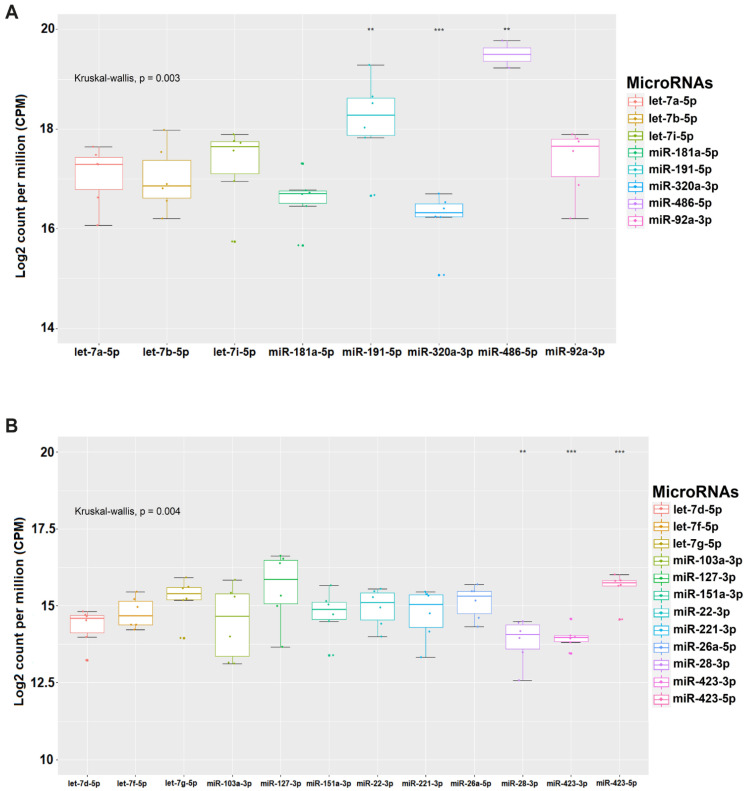
Different measures of expression level in PCs (**A**) and (**B**) with the normalized expression level in Log_2_ (CPM) of the 20 miRNAs most expressed. The boxplot is designed from the 75th to the 25th percentile. The vertical lines above and below the box define the maximum and minimum values and the dots indicate outliers, the horizontal line inside the box represents the median value. The Kruskal–Wallis test *p*-value < 0.001, in all cases was applied to compare the means among the miRNAs expressed in all PCs (** *p* < 0.01 and *** *p* = 0.001). Two groups of miRNAs were established to compare the relative expression by the analytical method previously mentioned to evaluate the PC bags.

In all PCs, high levels of expression of miR-486-5p and miR-191-5p were identified, let-7i-5p being the third most expressed miRNA until PC-3, followed by miR-92a-3p, miR-181a-5p, and the other miRNAs that also occupied prominent positions listed in Table 2. We show the ranking of miRNAs with the normalized expression on the Log_2_ CPM scale in two separate groups in the graphs depicted in Figure 2A,B.

The first group is represented by (miR-486-5p, miR-191-5p, miR-320a-3p, miR-181a-5p, let-7i-5p, let-7b-5p, let-7a-5p, and miR-92a-3p) and these miRNAs showed a range of expression between 16 and 20 CPM (Figure 2A). Whereas the second group (miR-127-3p, miR-423-5p, miR-26a-5p, let-7g-5p, miR-423-3p, miR-28-3p, let-7d-5p, miR-103a-3p, miR-221-3p, miR-151a-3p, let-7f-5p, and miR-22-3p) varied with expression values above 12.5, approaching 17.5 CPM (Figure 2B).

These two groups of miRNAs had their levels of expression analyzed by the analytical method which compares the relative expression of miRNAs identified by Pontes et al. In a PC bag we can test the following:

i. Any of the following miRNAs (miR-127-3p, miR-423-5p, miR-26a-5p, let-7g-5p, miR-423-3p, miR-28-3p, let-7d-5p, miR-103a-3p, miR-221-3p, miR-151a-3p, let-7f-5p, and miR-22-3p) that have an expression level <80% in relation to one of the following (miR-486-5p, miR-191-5p, miR-320a-3p, miR-181a-5p, let-7i-5p, let-7b-5p, let-7a-5p, or miR-92a-3p), means that there is storage lesion and immediate blockage of the bag that can be tested at any time.
ii. If in a PC bag the expression levels of (miR-127-3p, miR-423-5p, miR-26a-5p, let-7g-5p, miR-423-3p, miR-28-3p, let-7d-5p, miR-103a-3p, miR-221-3p, miR-151a-3p, let-7f-5p, and miR-22-3p) ≥80 in relation to one of the following (miR-486 -5p, miR-191-5p, miR-320a-3p, miR-181a-5p, let-7i-5p, let-7b-5p, let-7a-5p, or miR-92a-3p). It is considered that the PC bag can be used for transfusion, as there are no storage lesions.

**Figure 3:**
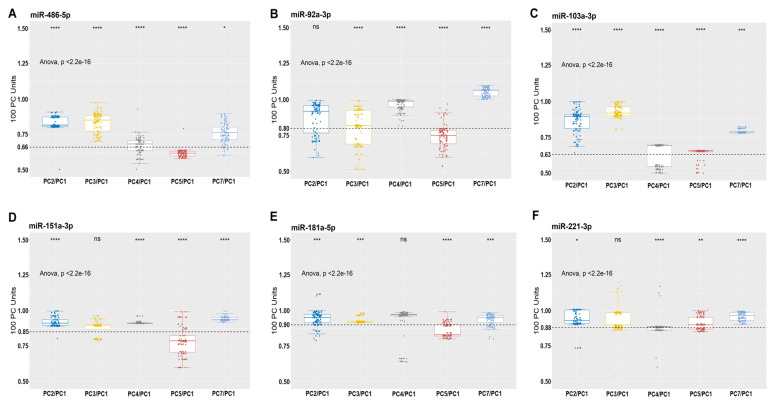
Relative mean expression of miRNAs (quantitative PCR (qPCR) analysis). The average expression of miR-486-5p, miR-92a-3p, miR-103a-3p, miR-151a-3p, miR-181a-5p, and miR-221-3p decreased from PC-4 to PC-5 and increased only on PC-7. ANOVA was applied in multiple comparison tests to estimate the significance of the relative mean of miRNAs in 100 PC units. (ns, not significant, * *p* < 0.05, ** *p* < 0.01, *** *p* = 0.001, **** *p* < 0.0001). In all graphs, the X-axis represents the storage time of the PCs, and the Y-axis represents the 100 PC units.

From the 20 most expressed miRNAs on the PCs, we selected six, i.e., miR-486-5p, miR-92a-3p, miR-103a-3p, miR-151a-3p, miR-181a-5p, and miR-221-3p for qPCR validation on 100 PC units from 500 healthy donors (Figure 3). These miRNAs were chosen among the 20 most expressed RNAs according to the following criteria: have been expressed in all PCs and have not been described in studies of platelet storage lesions. Relative quantification confirmed the results obtained with sRNA-Seq.

Our results show that miR-486-5p, miR-92a-3p, miR-103a-3p, miR-151a-3p, miR-181a-5p, and miR-221-3p decrease from the fourth to the fifth day of PC storage (Figure 3). We compared the average expression of these miRNAs in 100 units of PC on the fourth day (PC-4), confirming that the storage time caused the decrease of these miRNAs, in a much more accentuated way for miR-486-5p more expressed in the sRNA-Seq data (Table 2). All six miRNAs increased their levels of expression on PC-7 (Figure 3).

**Figure 4:**
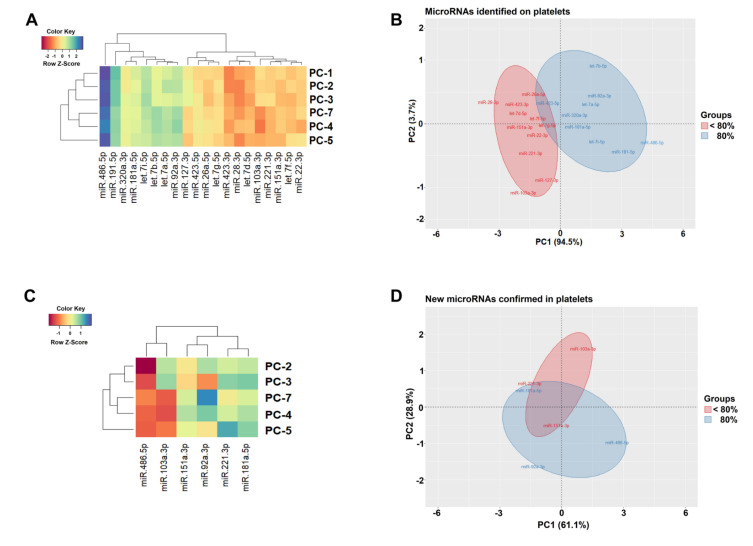
Hierarchical cluster (**A**) and principal component analysis (PCA) (**B**), generated with sRNA-Seq data. Hierarchical cluster (**C**) and PCA (**D**) generated with the validation data by qPCR.

To understand how the increased of storage time caused changes in miRNAs profiles, we applied computational biology simulation to sRNA-Seq and qPCR data. The results showed miRNAs grouped with similar expression profiles, both for increasing and decreasing expression (Figure 4A,C), and these miRNAs, most likely, could or could not have the same function in PCs with activated platelets. The PCA analysis (Figure 4B,D) was able to identified MiRNAs groups that suffered expression variation caused by the increased storage time, as we observed miR-486-5p, miR-92a-3p, and miR-151a-3p at extreme points of the ellipses and miR-103a-3p, miR-181a-5p, and miR-221-3p (overlapping ellipses). The first principal components (PC1) explained 64.1% of the total miRNA expression variations in the qPCR experiment (Figure 4D).

The hierarchical grouping (Figure 4A) shows an expression pattern of 20 miRNAs in six PCs identified with sRNA-Seq. Z-score was the metric applied to infer miRNAs with similar levels of expression. Gradients with a red tendency represent miRNAs with a lower Z-score and gradients with a blue tendency with a higher Z-score. The PCA analysis graph (Figure 4B) shows the grouping of miRNAs with expression level <80% (in red, according to the miR-127-3p expression reference) and miRNAs with expression level 80% on the PC (in blue, according to the miR-320a-3p expression reference). The hierarchical cluster (Figure 4C) and PCA (Figure 4D) identify the associations of miRNAs, miR-486-5p, miR-92a-3p, miR-103a-3p, miR-151a-3p, miR-181a-5p, and miR-221-3p validated with qPCR related to storage lesions. In the PCA analysis graph, ellipses were predicted with a probability of 0.95. The X- and Y-axes show principal component 1 and principal component 2. The first principal component (PC1), explained 94.5% and 64.1% of the total miRNA expression variations in the two experiments, respectively. In both PCAs, the divergences in the first two main components reflect the differences in miRNA profiles with a particularly distinct division between groups.

### 3.3. IsomiR Quantification

**Figure 5:**
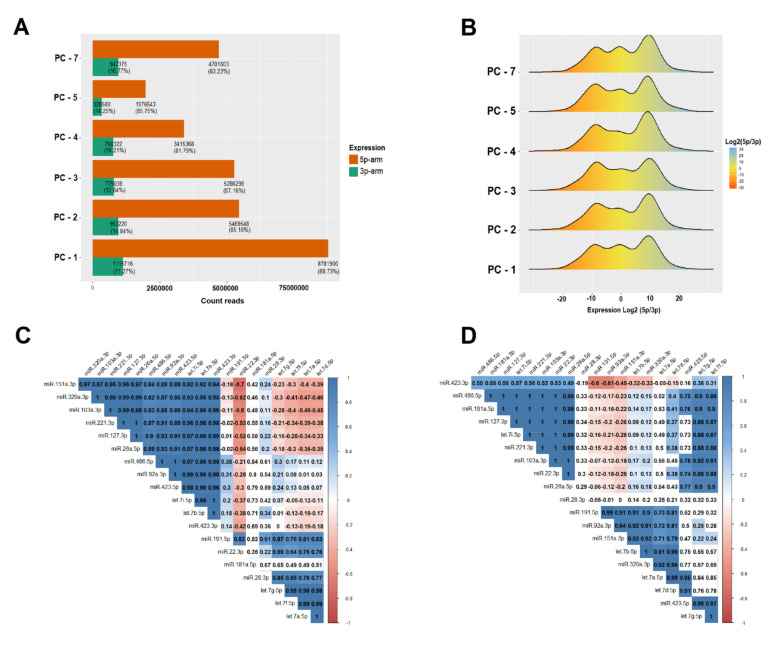
(**A**) Expression pattern of miRNAs in 5p-arm and 3p-arm. We showed the amount of crude and (%) count read expressed in the 5p-arm and 3p-arm that are estimated in all PCs; (**B**) Density of miRNA expression of each PC measured by the log_2_ of the ratio (5p-arm/3p-arm); (**C**) Correlogram calculated to highlight the miRNAs that are most correlated with the NTA variants, with an emphasis on miR-486-5p, miR-92a-3p, miR-320a-3p, miR-127-3p, let-7i -5p, let-7b-5p, miR-103a-3p, miR-151a-3p, miR-221-3p, miR-26a-5p, miR-423-3p, and miR-423-5p; (**D**) Correlogram calculated correlation to highlight the miRNAs that most correlate with the length variants, highlighting miR-486-5p, miR-127-3p, let-7i-5p, miR-103a-3p, miR-423-3p, miR-181a-5p, miR-22-3p, let-7d-5p, miR-423-5p, and let-7g-5p. MiRNAs that have a positive correlation for both variants at the same time were miR-486-5p, miR-127-3p, let-7i-5p, miR-103a-3p, miR-423-3p, and miR-423-5p. In the correlograms, the scales with blue gradient are positively correlated, while the gradients in red color are negatively correlated. Blank scale are miRNAs correlations with *p*-value >0.01 are considered no significant.

We identified a dominant pattern of expression of mature miRNAs in 5p-arm and 3p-arm, which were investigated systematically. Our results show that non-activated platelets (PC-1), present more than 88.73% of specific miRNAs in 5p-arm and only 11.27% in 3p-arm (Figure 5A). We extended this count across all PCs in an attempt to find significant differences in 5p-arm and 3p-arm expression dominance.

The data indicate that the percentage decrease in miRNA, caused by storage, was 85.75% in 5p-arm and 14.25% in 3p-arm (PC-5) as compared with previous PCs (Figure 5A). We measured the density of miRNA expression by the log_2_ ratio (5p-arm/3p-arm). A significant difference was found in the level of expression, tens, or hundreds of times greater in the 5p-arm than in the 3p-arm (Figure 5B).

A large number of miRNA different sequences variants were identified in all PCs in a reproducible way (Table 3). In Table 3(1) we identified the total of isomiR expressed in count reads in the form of NTA and length variants. In Table 3(2) we account for the mean and standard deviation of these variants in count reads, detected in the canonical sequence of the 20 miRNAs. The averages were tested with ANOVA (*p* < 0.001) and were significant. On the one hand, the amount of the NTA(U) variant increased from PC-1 to PC-7 to 67.31%, on the other hand, the NTA(A) variant decreased from PC-1 to PC-7 to 30.42%. The variants NTA(C) and NTA(G) showed less relevant variations in increase and decrease.

The most abundant length variants were lv3pT which decreased from PC-1 to PC-7 to 63.64%, whereas lv5pE increased from PC-1 to PC-7 to 30.11%. The lv5pE, lv5pT, and mv variants were less abundant. Sequence variants were correlated to miRNAs by calculating the correlation matrix (Figure 5C,D), which highlighted the following miRNAs: miR-486-5p, miR-127-3p, let-7i-5p, miR-103a-3p, miR-423-3p, and miR-423-5p (Table 3). These showed a positive correlation for both variants at the same time. We gathered more detailed information on these variants which is available in Supplementary Materials File S2.

### 3.4. Functional microRNA-gene Interaction on the PC

**Figure 6:**
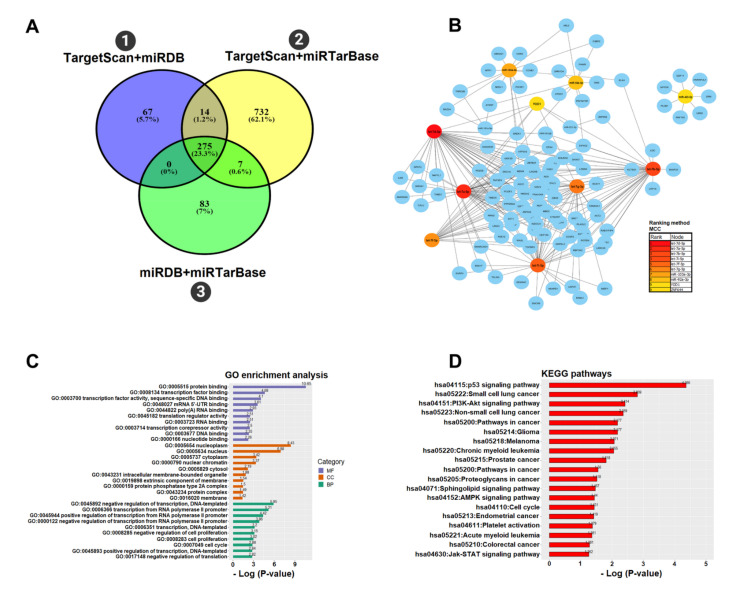
(**A**) Three subsets of data obtained with target prediction with miRWalk generated 67 (5.7%) interactions in file (1) TargetScan + miRDB, 732 (62.1%) of interactions in file (2) TargetScan + miRTarBase and 83 (7%) of interactions in file (3) miRDB + miRTarBase. Both files share 275 (23.3%) interactions at the same time; (**B**) The network of miRNA–gene interactions built with Cytoscape using the maximal clique centrality (MCC) method that was applied to identify regions of denser connections of miRNA–gene interactions. The nodes are represented by the red, orange, and yellow gradient circles that were ranked by the MCC. The edges are identified by circles in blue; (**C**) The most enriched GO terms in three functional categories, i.e., molecular function (MF), cell component (CC), and biological process (BP) for the main genes of the network constructed from 275 miRNA–gene interactions; (**D**) The main KEGG pathways for the functional enrichment of genes in this network were normalized with -Log_10_ (*p*-value).

The results of the miRNAs target prediction genes showed that the subsets of predicted and validated interactions share 275 (23.3%) of accounted interactions (Figure 6A). The first network of miRNA–gene interaction constructed with file (1), presented 339 nodes and 1070 edges, while the second network constructed with file (2) presented 127 nodes and 560 edges. The final network merged from the first two networks presents 108 nodes and 220 edges (Figure 6B). The network topology presents a central region formed by denser connections with miRNA–gene interactions, ordered by the MCC mainly for let-7d-5p, let-7a-5p, let-7i-5p, let-7b-5p, let-7f-5p, and let-7g-5p, in addition to the *YOD1* gene and compositions formed by miR-92a-3p, miR-423-5p and miR-103a-3p.

Most of the genes in the network in Figure 6B were enriched in GO, mainly in the categories of molecular function for protein binding (GO: 0005515), cellular component for nucleoplasm (GO: 0005654), and in a biological process for negative regulation of transcription, DNA-templated (GO: 0045892) (Figure 6C). These genes were also enriched with DAVID [39], generating a wide pathway panel with an important functional repertoire for the *P53* signaling pathway, described in signs of oxidative stress, including DNA damage, activation of oncogenes, cell cycle arrest, senescence, and apoptosis. Pathways with an impact on cancer, cell cycle and platelet activation, and other pathways directly associated with cellular stimuli, signal transduction, cell signaling, and stress response were predicted (Figure 6D). The results of these analyses are in Supplementary Material file S3.

## 4. Discussion

Aging is characterized by a functional decline in many physiological systems that can be triggered by environmental and endogenous stress, including the wear on telomeres, genomic instability, epigenetic changes, and loss of proteostasis, favoring cell damage and the progression of physiological aging [43].

Platelets are small anucleated cells that essentially originate from the fragmentation of pseudopods from the megakaryocyte cytoplasmic membrane in the bone marrow [44]. In a healthy person, platelets circulate in the blood for about seven to ten days, and then are removed from circulation and destroyed in the spleen [45,46]. Similarly, this aging also occurs in vitro, wherein blood banks in most countries, blood components widely used and routinely supplied in the form of PCs are discarded after five days of storage [47].

Platelet storage in blood banks causes a decrease in the abundance of miRNAs due to shear stress and platelet activation, being the two main factors responsible for the release of microparticles (MPs) rich in miRNAs [11,12,13]. Studies of this nature have shown a relationship between miRNA profiles with subsequent platelet reactivity, suggesting an important role in post-transcriptional regulation during storage [7,24,48].

In this study, we identified the 20 most expressed miRNAs in PCs stored for seven days with a genomic coverage of 95%. Specifically, in the PC with high-quality platelets (PC-1), we identified a total of 916 miRNAs (Table 1). However, we confirmed a 22.4% decrease in miRNA levels from the first to the fifth day, much more accentuated than the value found in our previous study [20], increasing the number of miRNAs only after seven days of blood collection with a rate of 2.5, which is, most likely, a response to inhibit the translation of proteins induced by stress caused by aging for more than seven days of storage [20]. On the basis of these results, we emphasize that the use of different bioinformatics pipelines to analyze the same sequencing data, generates results that can differ substantially. Whereas, some studies have highlighted the urgent need to ensure that the bioinformatics pipelines used for next-generation sequencing (NGS) analysis, undergo better validation, especially for applications in translational genomic medicine [49].

In our data, we observed the influence of storage on the unequal distribution of the abundance of miRNA families in all PCs (Figure 1). For example, the largest mir-486 family showed declines in expression, losing the most abundant position on PC-3 to the mir-423 family. The mir-191 family was replaced by members of the let-7 family on PC-2, which are very common in platelets [50]. Probably, post-transcriptional changes influenced the biogenesis and stability of miRNA during storage, as has been shown in studies that used molecular changes in DICER1 to reduce the number of miRNAs that strongly regulated platelet reactivity [51,52].

The measurement of miRNAs expression in the PCs is a variable that we have demonstrated to be associated with the quality of these blood components. When we evaluated the expression levels of miRNA in PCs using computational methodologies, we concluded that, in clinical practice, miRNAs are a very useful tool for testing PC bags that are close to expiration date during storage in a blood bank.

In this study, we found a large number of miRNAs that are candidates as storage damage biomarkers that can replace miR-127, miR-191, and miR-320a miRNAs in clinical trials, which were found in our first study, especially miR-191 which has been used as an internal control for qPCR validation analysis [20].

In addition, we selected six new miRNAs, of the most expressed RNAs, for further validation by qPCR. Relative quantification indicates decreased expression levels of miR-486-5p, miR-92a-3p, miR-103a-3p, miR-151a-3p, miR-181a-5p, and miR-221-3p from the fourth to the fifth day (Figure 3). We also highlighted that this decrease occurred more accentuated for miR-486-5p, which was more expressed in the sRNA-Seq data (Table 2). Additionally, all six miRNAs increased their levels of expression on PC-7 (Figure 3).

We applied computational biology simulations (hierarchical grouping and PCA analysis) to the data generated by sRNA-Seq and qPCR which revealed how the increase in platelet storage time caused changes in the miRNA profiles confirmed in the validation. These computational methodologies have resulted in more accurately identifying miRNAs located in different groups based on the days of storage [53]. In our previous study, we used these methodologies which identified changes in the expression profiles of 14 miRNAs that were associated with PSL [21]. In this current study, we confirmed that changes in the profiles of the new miRNAs correlated with the instability of the half-life of these transcripts on the fourth day, which coincided with the time of onset of PSL. For example, PC bags that are tested and confirm that the expression of miRNAs (miR-151a-3p, miR-103a-3p, and miR-221-3p) is <80% of the expression of (miR-486-5p, miR-92a-3p, and miR-181a-5p) means that there are storage lesions.

The miRNA’s stability varies widely, with half-lives of ~1.5 h, more than 13 h, and up to 48 h, in human biofluids [54,55]. Measuring the relative levels of miRNA in PC is subject to some challenges that need to be taken into account, because the relative stability of miRNAs has implications for their ability to transfer regulatory information, as they are very short and have highly divergent sequences, with a wide variation of the GC content that can favor the different hybridization properties among different miRNA sequences [55].

In a blood bank, the analytical method for testing PCs can be implemented quickly and with low cost to test PC bags stored for more than four days whihc still contain physiologically normal platelets. The durability of platelet physiology depends on individuals with ideal suboptimal health status (SHS), which is considered to be a subclinical and reversible stage of chronic disease. Individuals with SHS can have a progressive accumulation of senescent cells and a relative shortening of the telomeres that produces the early biological aging of platelets [56,57,58].

The increase of miRNA levels in the PCs suggests that after platelet activation they stabilize within the circulating MPs for their transport of action [7,13,59], undergoing changes in their profiles and using selective platelet packaging pathway for MPs [13,60]. For example, we justify expression changes on several PCs, based on the dominant expression pattern in 5p-arm about 3p-arm (Figure 5B). The density measurement confirmed a significant difference in the level of expression, tens or hundreds of times greater in the 5p-arm than in the 3p-arm, as has been observed in other studies [61,62,63].

Our global estimates of miRNAs expression on all PCs pointed to a decline in this dominant pattern from PC-2 to PC-5, but only increased on PC-7 (Table 3). We found several non-model and length variants, positively correlated with recently emerged miRNAs (miR-486-5p, miR-92a-3p, miR-103a-3p, miR-151a-3p, miR-181a-5p, and miR-221-3p), which have not yet been reported in other platelet miRNomes (complete sequencing of miRNAs) and provide greater quality and innovation to the analytical test mentioned in that study.

Our analysis of miRNA–gene functional interaction, pointed to the existence of a molecular regulation mechanism in platelets, which was inferred based on the topology of the interaction network formed by recently emerged miRNAs (miR-103a-3p, miR-423-5p, and miR-92a-3p) and conserved miRNAs of the let-7 family interacting with the *YOD1* gene, a desubiquitinating enzyme that is very expressed in platelet hyperactivity [56]. We also identified the functional roles of significant target genes in signaling pathways, cell cycle, stress response, platelet activation, and cancer.

A limitation of this study is the relatively small number of miRNAs investigated, only 20 more expressed, among which, some have been intensively reviewed by the literature [7,30,64,65,66]. Our results also provided a panel of miRNAs that have had their functions elucidated in platelets such as miR-223 [24,66], miR-20a [23] miR-126 [67], miR-10a/miR-10b [68], miR-326 [69], miR-150/miR-501/miR-338-5p/miR-432-5p/miR-411-5p [21], miR-570 [18], miR-495 [70], and miR-21/miR-27b [71]. For example, specific cold storage conditions caused changes in miRNA expressions, increasing levels of miR-20a/miR-10a/miR-16-2/miR-223, which correlated with the quality of platelets under specific conditions of storage [23]. Other miRNAs have been found to play an important role in platelet apoptosis stored as miR-326/miR-570 [18]. Increased expression of miR-21 and miR-27b were found in activated platelet microparticles caused by cooling [71]. In addition to these, there are a variety of new miRNAs that have not yet been reported in the literature (Supplementary Materials S2).

In the future, our investigations should be repeated in a larger number of samples, studying the sixth day of storage (not carried out with the current data). Fresh platelets (PC-0) should also be studied to obtain a broader profile of expression variation with storage days. The option to extend the study after seven days of storage would also be an advantage.

In summary, our results have a promising application in transfusion medicine, because we describe a new collection of miRNAs (miR-486-5p, miR-92a-3p, miR-103a-3p, miR-151a-3p, miR-181a-5p, and miR-221-3p) that shows a sensitivity expression pattern due to biological platelet changes during storage. These miRNAs could be applied, in blood banks, as potential biomarkers to also measure the quality and viability of the PC during storage.

## Figures and Tables

**Table 1:** A summary of microRNA (miRNA) analysis in platelet concentrates.

Preprocessing Summary (PC-Day) SRA:	PC-1	PC-2	PC-3	PC-4	PC-5	PC-7
	SRX716593	SRX716594	SRX716595	SRX716596	SRX716597	SRX716598
Raw input reads	16,212,635	13,594,963	17,214,842	25,325,847	16,161,626	28,515,834
Trimmed reads	16,112,769 (99.38%)	13,499,180 (99.29%)	17,101,122 (99.33%)	25,093,371 (99.08%)	16,034,860 (99.21%)	28,280,300 (99.17%)
Reads in analysis	15,456,133 (95.33%)	12,732,995 (93.65%)	16,564,900 (96.22%)	24,291,441 (95.91%)	15,173,571 (93.88%)	26,665,506 (93.51%)
Genome/Library mapping	
Unique genome mapped reads	83,673 (83.21%)	94,716 (83.32%)	129,731 (85.26%)	219,742 (81.06%)	176,766 (85.44%)	256,859 (82.18%)
Genome mapped reads	14,685,047 (95.01%)	11,757,300 (92.34%)	14,969,574 (90.37%)	22,681,622 (93.37%)	13,734,766 (90.52%)	24,321,990 (91.21%)
MiR profiling results	
*^•^* Reads mapped to miRbase hairpins	5498 (0.09%)	5509 (0.13%)	6896 (0.16%)	6232 (0.21%)	4500 (0.26%)	13,042 (0.30%)
Detected hairpin miR	610 (31.82%)	567 (29.58%)	562 (29.32%)	574 (29.94%)	486 (25.35%)	644 (33.59%)
*^•^* Reads mapped to mature miR	6,042,081 (96.83%)	3,997,760 (95.42%)	3,812,542 (89.64%)	2,643,351 (88.88%)	1,420,368 (83.35%)	3,586,353 (81.90%)
Detected mature miRNA	916 (34.49%)	842 (31.70%)	834 (31.40%)	825 (31.06%)	711 (26.77%)	939 (35.35%)

***^•^*** RC, reads count of the mature sequence (canonical sequence and all isomiRs). MiRNA reference miRBase, version 22 (http://www.mirbase.org). Hairpins, 1917 and mature, 2656.

**Table 2:** The top-20 miRNAs most expressed in platelet concentrates.

(1) Pre-microRNA								(2) Mature microRNA					
	Gene Family	Coordinate String	Pre-microRNA	Unique Reads	Read Count (RC)	RPM (Lib)	RPM (Total)	Mature microRNA	Sequence: miR -5p/-3p Arms	Unique Reads	Read Count (RC)	RPM (Lib)	RPM (Total)
**PC-1 (SRX716593)**	mir-486	8:hsa;mir-486-2,41660444,41660465,+	mir-486-2	1292	3,255,194	538,264	221,667	miR-486-5p	5p-UCCUGUACUGAGCCGCCCCGAG	1189	3,253,869	538,534	221,577
	mir-191	3:hsa;mir-191,49020633,49020655,-	mir-191	335	639,518	105,748	43,549	miR-191-5p	5p-CAACGGAAUCCCAAAAGCAGCUG	318	639,420	105,828	43,542
	let-7	12:hsa;let-7i,62603691,62603712,+	let-7i	205	243,758	40,307	16,599	let-7i-5p	5p-UGAGGUAGUAGUUUGUGCUGUU	194	243,687	40,332	16,594
	mir-25	13:hsa;mir-92a-1,91351361,91351382,+	mir-92a-1	510	220,091	36,393	14,987	miR-92a-3p	3p-UAUUGCACUUGUCCCGGCCUGU	497	220,034	36,417	14,984
	mir-181	1:hsa;mir-181a-1,198859067,198859089,-	mir-181a-1	264	161,979	26,784	11,030	miR-181a-5p	5p-AACAUUCAACGCUGUCGGUGAGU	254	161,722	26,766	11,013
	let-7	9:hsa;let-7a-1,94175962,94175983,+	let-7a-1	175	159,936	26,446	10,891	let-7a-5p	5p-UGAGGUAGUAGGUUGUAUAGUU	173	159,928	26,469	10,891
	let-7	22:hsa;let-7b,46113691,46113712,+	let-7b	304	121,975	20,169	8306	let-7b-5p	5p-UGAGGUAGUAGGUUGUGUGGUU	286	121,845	20,166	8297
	mir-320	8:hsa;mir-320a,22245007,22245028,-	mir-320a	578	86,702	14,337	5904	miR-320a-3p	3p-AAAAGCUGGGUUGAGAGGGCGA	561	86,637	14,339	5900
	mir-127	14:hsa;mir-127,100883035,100883056,+	mir-127	320	84,993	14,054	5788	miR-127-3p	3p-UCGGAUCCGUCUGAGCUUGGCU	283	84,810	14,037	5775
	mir-103	20:hsa;mir-103a-2,3917541,3917563,+	mir-103a-2	188	58,511	9675	3984	miR-103a-3p	3p-AGCAGCAUUGUACAGGGCUAUGA	181	58,469	9677	3982
	mir-26	12:hsa;mir-26a-2,57824622,57824643,-	mir-26a-2	132	52,776	8727	3594	miR-26a-5p	5p-UUCAAGUAAUCCAGGAUAGGCU	131	52,774	8734	3594
	mir-28	8:hsa;mir-151a,140732610,140732630,-	mir-151a	274	63,281	10,464	4309	miR-151a-3p	3p-CUAGACUGAAGCUCCUUGAGG	221	51,561	8534	3511
	mir-423	17:hsa;mir-423,30117095,30117117,+	mir-423	433	66,631	11,018	4537	miR-423-5p	5p-UGAGGGGCAGAGAGCGAGACUUU	213	51,007	8442	3473
	let-7	3:hsa;let-7g,52268282,52268303,-	let-7g	114	48,462	8013	3300	let-7g-5p	5p-UGAGGUAGUAGUUUGUACAGUU	110	48,451	8019	3299
	mir-22	17:hsa;mir-22,1713955,1713976,-	mir-22	88	48,146	7961	3279	miR-22-3p	3p-AAGCUGCCAGUUGAAGAACUGU	81	48,015	7947	3270
	mir-10	X:hsa;mir-221,45746221,45746243,-	mir-221	190	45,199	7474	3078	miR-221-3p	3p-AGCUACAUUGUCUGCUGGGUUUC	168	44,952	7440	3061
	mir-221	X:hsa;let-7f-2,53557199,53557220,-	let-7f-2	95	38,034	6289	2590	let-7f-5p	5p-UGAGGUAGUAGAUUGUAUAGUU	94	38,032	6295	2590
	let-7	9:hsa;let-7d,94178841,94178862,+	let-7d	125	29,204	4829	1989	let-7d-5p	5p-AGAGGUAGUAGGUUGCAUAGUU	88	25,688	4252	1749
	let-7	3:hsa;mir-28,188688834,188688855,+	mir-28	154	23,667	3913	1612	miR-28-3p	3p-AAGGAGCUCACAGUCUAUUGAG	134	23,057	3816	1570
	mir-423	17:hsa;mir-423,30117131,30117153,+	mir-423	433	66,631	11,018	4537	miR-423-3p	3p-AGCUCGGUCUGAGGCCCCUCAGU	216	15,610	2584	1063
**PC-2 (SRX716594)**				6209	5,514,688	911,884	375,531			5392	5,429,568	898,626	369,734
	mir-486	8:hsa;mir-486-2,41660444,41660465,+	mir-486-2	1048	1,706,986	426,398	145,185	miR-486-5p	5p-UCCUGUACUGAGCCGCCCCGAG	995	1,705,861	426,704	145,090
	mir-191	3:hsa;mir-191,49020633,49020655,-	mir-191	264	412698	103,090	35,101	miR-191-5p	5p-CAACGGAAUCCCAAAAGCAGCUG	251	412,617	103,212	35,095
	let-7	12:hsa;let-7i,62603691,62603712,+	let-7i	206	216,034	53,964	18,374	let-7i-5p	5p-UGAGGUAGUAGUUUGUGCUGUU	195	215,993	54,029	18,371
	let-7	9:hsa;let-7a-1,94175962,94175983,+	let-7a-1	191	204,590	51,106	17,401	let-7a-5p	5p-UGAGGUAGUAGGUUGUAUAGUU	186	204,568	51,171	17,399
	mir-25	13:hsa;mir-92a-1,91351361,91351382,+	mir-92a-1	476	193,534	48,344	16,461	miR-92a-3p	3p-UAUUGCACUUGUCCCGGCCUGU	465	193,492	48,400	16,457
	let-7	22:hsa;let-7b,46113691,46113712,+	let-7b	305	115,041	28,737	9785	let-7b-5p	5p-UGAGGUAGUAGGUUGUGUGGUU	286	114,919	28,746	9774
	mir-181	1:hsa;mir-181a-1,198859067,198859089,-	mir-181a-1	227	112,339	28,062	9555	miR-181a-5p	5p-AACAUUCAACGCUGUCGGUGAGU	220	112,168	28,058	9540
	mir-127	14:hsa;mir-127,100883035,100883056,+	mir-127	358	100,453	25,093	8544	miR-127-3p	3p-UCGGAUCCGUCUGAGCUUGGCU	325	100,285	25,085	8530
	mir-320	8:hsa;mir-320a,22245007,22245028,-	mir-320a	563	77,271	19,302	6572	miR-320a-3p	3p-AAAAGCUGGGUUGAGAGGGCGA	549	77,217	19,315	6568
	let-7	3:hsa;let-7g,52268282,52268303,-	let-7g	114	61,357	15,327	5219	let-7g-5p	5p-UGAGGUAGUAGUUUGUACAGUU	114	61,357	15,348	5219
	mir-423	17:hsa;mir-423,30117095,30117117,+	mir-423	429	68,554	17,125	5831	miR-423-5p	5p-UGAGGGGCAGAGAGCGAGACUUU	230	52,321	13,088	4450
	mir-221	X:hsa;let-7f-2,53557199,53557220,-	let-7f-2	104	44,961	11,231	3824	let-7f-5p	5p-UGAGGUAGUAGAUUGUAUAGUU	103	44,959	11,246	3824
	mir-103	20:hsa;mir-103a-2,3917541,3917563,+	mir-103a-2	173	43,831	10,949	3728	miR-103a-3p	3p-AGCAGCAUUGUACAGGGCUAUGA	169	43,806	10,958	3726
	mir-10	X:hsa;mir-221,45746221,45746243,-	mir-221	165	42,299	10,566	3598	miR-221-3p	3p-AGCUACAUUGUCUGCUGGGUUUC	155	42,203	10,557	3590
	mir-22	17:hsa;mir-22,1713955,1713976,-	mir-22	80	39,426	9848	3353	miR-22-3p	3p-AAGCUGCCAGUUGAAGAACUGU	76	39,365	9847	3348
	mir-26	12:hsa;mir-26a-2,57824622,57824643,-	mir-26a-2	102	36,592	9141	3112	miR-26a-5p	5p-UUCAAGUAAUCCAGGAUAGGCU	102	36,592	9153	3112
	mir-28	8:hsa;mir-151a,140732610,140732630,-	mir-151a	223	42,658	10,656	3628	miR-151a-3p	3p-CUAGACUGAAGCUCCUUGAGG	173	33,633	8413	2861
	let-7	9:hsa;let-7d,94178841,94178862,+	let-7d	128	33,592	8391	2857	let-7d-5p	5p-AGAGGUAGUAGGUUGCAUAGUU	88	28,835	7213	2453
	mir-423	17:hsa;mir-423,30117131,30117153,+	mir-423	429	68,554	17,125	5831	miR-423-3p	3p-AGCUCGGUCUGAGGCCCCUCAGU	194	16,218	4057	1379
	let-7	3:hsa;mir-28,188688834,188688855,+	mir-28	125	16,257	4061	1383	miR-28-3p	3p-AAGGAGCUCACAGUCUAUUGAG	112	15,755	3941	1340
**PC-3 (SRX716595)**				5710	3,637,027	908,514	309,342			4988	3,552,164	888,539	302,124
	mir-486	8:hsa;mir-486-2,41660444,41660465,+	mir-486-2	370	120,353	31,511	8040	miR-486-5p	5p-UCCUGUACUGAGCCGCCCCGAG	982	1,894,623	496,945	126,565
	mir-191	3:hsa;mir-191,49020633,49020655,-	mir-191	260	374,338	98,009	25,007	miR-191-5p	5p-CAACGGAAUCCCAAAAGCAGCUG	249	374,253	98,164	25,001
	let-7	12:hsa;let-7i,62603691,62603712,+	let-7i	186	221,365	57,957	14,788	let-7i-5p	5p-UGAGGUAGUAGUUUGUGCUGUU	180	221,341	58,056	14,786
	mir-25	13:hsa;mir-92a-1,91351361,91351382,+	mir-92a-1	476	193,534	48,344	16,461	miR-92a-3p	3p-UAUUGCACUUGUCCCGGCCUGU	357	120,306	31,555	8037
	mir-181	1:hsa;mir-181a-1,198859067,198859089,-	mir-181a-1	219	106,232	27,814	7097	miR-181a-5p	5p-AACAUUCAACGCUGUCGGUGAGU	212	106,081	27,824	7086
	let-7	9:hsa;let-7a-1,94175962,94175983,+	let-7a-1	138	100,930	26,425	6742	let-7a-5p	5p-UGAGGUAGUAGGUUGUAUAGUU	136	100,925	26,472	6742
	let-7	22:hsa;let-7b,46113691,46113712,+	let-7b	267	96,496	25,264	6446	let-7b-5p	5p-UGAGGUAGUAGGUUGUGUGGUU	254	96,409	25,287	6440
	mir-320	8:hsa;mir-320a,22245007,22245028,-	mir-320a	605	94,553	24,756	6316	miR-320a-3p	3p-AAAAGCUGGGUUGAGAGGGCGA	588	94,482	24,782	6312
	mir-127	14:hsa;mir-127,100883035,100883056,+	mir-127	348	93,636	24,516	6255	miR-127-3p	3p-UCGGAUCCGUCUGAGCUUGGCU	320	93,488	24,521	6245
	mir-423	17:hsa;mir-423,30117095,30117117,+	mir-423	1053	1,895,916	496,386	126,651	miR-423-5p	5p-UGAGGGGCAGAGAGCGAGACUUU	217	57,556	15,096	3845
	mir-10	X:hsa;mir-221,45746221,45746243,-	mir-221	173	41,049	10,747	2742	miR-221-3p	3p-AGCUACAUUGUCUGCUGGGUUUC	161	40,980	10,749	2738
	mir-103	20:hsa;mir-103a-2,3917541,3917563,+	mir-103a-2	162	40,194	10,524	2685	miR-103a-3p	3p-AGCAGCAUUGUACAGGGCUAUGA	156	40,167	10,535	2683
	let-7	3:hsa;let-7g,52268282,52268303,-	let-7g	89	37,080	9708	2477	let-7g-5p	5p-UGAGGUAGUAGUUUGUACAGUU	88	37,076	9725	2477
	mir-22	17:hsa;mir-22,1713955,1713976,-	mir-22	72	31,563	8264	2108	miR-22-3p	3p-AAGCUGCCAGUUGAAGAACUGU	67	31,518	8267	2105
	mir-26	12:hsa;mir-26a-2,57824622,57824643,-	mir-26a-2	85	24,891	6517	1663	miR-26a-5p	5p-UUCAAGUAAUCCAGGAUAGGCU	85	24,891	6529	1663
	mir-28	8:hsa;mir-151a,140732610,140732630,-	mir-151a	184	27,165	7112	1815	miR-151a-3p	3p-CUAGACUGAAGCUCCUUGAGG	142	23,095	6058	1543
	mir-221	X:hsa;let-7f-2,53557199,53557220,-	let-7f-2	59	21,314	5580	1424	let-7f-5p	5p-UGAGGUAGUAGAUUGUAUAGUU	59	21,314	5591	1424
	mir-423	17:hsa;mir-423,30117131,30117153,+	mir-423	426	74,297	19,452	4963	miR-423-3p	3p-AGCUCGGUCUGAGGCCCCUCAGU	206	16,734	4389	1118
	let-7	9:hsa;let-7d,94178841,94178862,+	let-7d	107	20,065	5253	1340	let-7d-5p	5p-AGAGGUAGUAGGUUGCAUAGUU	69	16,136	4232	1078
	let-7	3:hsa;mir-28,188688834,188688855,+	mir-28	105	11,734	3072	784	miR-28-3p	3p-AAGGAGCUCACAGUCUAUUGAG	95	11,468	3008	766
**PC-4 (SRX716596)**				5384	3,626,705	947,212	245,804			4623	3,422,843	897,785	228,653
	mir-486	8:hsa;mir-486-2,41660444,41660465,+	mir-486-2	935	894,301	337,525	39,428	miR-486-5p	5p-UCCUGUACUGAGCCGCCCCGAG	873	893,294	337,940	39,384
	mir-191	3:hsa;mir-191,49020633,49020655,-	mir-191	241	231,963	87,547	10,227	miR-191-5p	5p-CAACGGAAUCCCAAAAGCAGCUG	231	231,904	87,731	10,224
	mir-25	13:hsa;mir-92a-1,91351361,91351382,+	mir-92a-1	564	229,695	86,691	10,127	miR-92a-3p	3p-UAUUGCACUUGUCCCGGCCUGU	551	229,650	86,878	10,125
	let-7	22:hsa;let-7b,46113691,46113712,+	let-7b	472	190,538	71,912	8401	let-7b-5p	5p-UGAGGUAGUAGGUUGUGUGGUU	403	190,076	71,907	8380
	let-7	9:hsa;let-7a-1,94175962,94175983,+	let-7a-1	201	160,740	60,666	7087	let-7a-5p	5p-UGAGGUAGUAGGUUGUAUAGUU	190	160,652	60,776	7083
	let-7	12:hsa;let-7i,62603691,62603712,+	let-7i	193	127,164	47,994	5606	let-7i-5p	5p-UGAGGUAGUAGUUUGUGCUGUU	182	127,135	48,096	5605
	mir-181	1:hsa;mir-181a-1,198859067,198859089,-	mir-181a-1	215	89,739	33,869	3956	miR-181a-5p	5p-AACAUUCAACGCUGUCGGUGAGU	200	89,565	33,883	3949
	mir-320	8:hsa;mir-320a,22245007,22245028,-	mir-320a	686	77,769	29,351	3429	miR-320a-3p	3p-AAAAGCUGGGUUGAGAGGGCGA	653	77,613	29,362	3422
	mir-423	17:hsa;mir-423,30117095,30117117,+	mir-423	440	71,225	26,882	3140	miR-423-5p	5p-UGAGGGGCAGAGAGCGAGACUUU	265	56,960	21,548	2511
	mir-26	12:hsa;mir-26a-2,57824622,57824643,-	mir-26a-2	118	45,261	17,082	1995	miR-26a-5p	5p-UUCAAGUAAUCCAGGAUAGGCU	118	45,261	17,123	1995
	let-7	3:hsa;let-7g,52268282,52268303,-	let-7g	104	38,106	14,382	1680	let-7g-5p	5p-UGAGGUAGUAGUUUGUACAGUU	101	38,097	14,412	1680
	mir-127	14:hsa;mir-127,100883035,100883056,+	mir-127	236	32,655	12,325	1440	miR-127-3p	3p-UCGGAUCCGUCUGAGCUUGGCU	210	32556	12,316	1435
	mir-28	8:hsa;mir-151a,140732610,140732630,-	mir-151a	214	32,928	12,428	1452	miR-151a-3p	3p-CUAGACUGAAGCUCCUUGAGG	171	26,728	10,111	1178
	let-7	9:hsa;let-7d,94178841,94178862,+	let-7d	135	30,214	11,403	1332	let-7d-5p	5p-AGAGGUAGUAGGUUGCAUAGUU	82	23,494	8888	1036
	let-7	3:hsa;mir-28,188688834,188688855,+	mir-28	136	22,513	8497	993	miR-28-3p	3p-AAGGAGCUCACAGUCUAUUGAG	125	22,352	8456	985
	mir-221	X:hsa;let-7f-2,53557199,53557220,-	let-7f-2	74	21,246	8019	937	let-7f-5p	5p-UGAGGUAGUAGAUUGUAUAGUU	72	21,241	8036	936
	mir-10	X:hsa;mir-221,45746221,45746243,-	mir-221	112	18,302	6908	807	miR-221-3p	3p-AGCUACAUUGUCUGCUGGGUUUC	105	18,262	6909	805
	mir-22	17:hsa;mir-22,1713955,1713976,-	mir-22	52	16,322	6160	720	miR-22-3p	3p-AAGCUGCCAGUUGAAGAACUGU	49	16,306	6169	719
	mir-423	17:hsa;mir-423,30117131,30117153,+	mir-423	440	71,225	26,882	3140	miR-423-3p	3p-AGCUCGGUCUGAGGCCCCUCAGU	166	14,240	5387	628
	mir-103	20:hsa;mir-103a-2,3917541,3917563,+	mir-103a-2	109	8830	3333	389	miR-103a-3p	3p-AGCAGCAUUGUACAGGGCUAUGA	108	8827	3339	389
**PC-5 (SRX716597)**				5677	2,410,736	909,855	106,286			4855	2,324,213	879,268	102,471
	mir-486	8:hsa;mir-486-2,41660444,41660465,+	mir-486-2	683	615,100	431,689	44,784	miR-486-5p	5p-UCCUGUACUGAGCCGCCCCGAG	652	614,560	432,677	44,745
	mir-191	3:hsa;mir-191,49020633,49020655,-	mir-191	157	104,250	73,165	7590	miR-191-5p	5p-CAACGGAAUCCCAAAAGCAGCUG	152	104,216	73,373	7588
	let-7	22:hsa;let-7b,46113691,46113712,+	let-7b	249	75,462	52,961	5494	let-7b-5p	5p-UGAGGUAGUAGGUUGUGUGGUU	230	75,389	53,077	5489
	mir-25	13:hsa;mir-92a-1,91351361,91351382,+	mir-92a-1	311	75,403	52,919	5490	miR-92a-3p	3p-UAUUGCACUUGUCCCGGCCUGU	305	75,383	53,073	5488
	let-7	9:hsa;let-7a-1,94175962,94175983,+	let-7a-1	123	68,853	48,322	5013	let-7a-5p	5p-UGAGGUAGUAGGUUGUAUAGUU	120	68,845	48,470	5012
	let-7	12:hsa;let-7i,62603691,62603712,+	let-7i	117	54,798	38,458	3990	let-7i-5p	5p-UGAGGUAGUAGUUUGUGCUGUU	113	54,789	38,574	3989
	mir-181	1:hsa;mir-181a-1,198859067,198859089,-	mir-181a-1	157	51,930	36,445	3781	miR-181a-5p	5p-AACAUUCAACGCUGUCGGUGAGU	147	51,833	36,493	3774
	mir-423	17:hsa;mir-423,30117131,30117153,+	mir-423	282	35,167	24,681	2560	miR-423-3p	3p-AGCUCGGUCUGAGGCCCCUCAGU	130	11,232	7908	818
	mir-423	17:hsa;mir-423,30117095,30117117,+	mir-423	282	35,167	24,681	2560	miR-423-5p	5p-UGAGGGGCAGAGAGCGAGACUUU	151	23,930	16,848	1742
	mir-320	8:hsa;mir-320a,22245007,22245028,-	mir-320a	377	34,546	24,245	2515	miR-320a-3p	3p-AAAAGCUGGGUUGAGAGGGCGA	367	34,507	24,294	2512
	mir-22	17:hsa;mir-22,1713955,1713976,-	mir-22	51	21,777	15,284	1586	miR-22-3p	3p-AAGCUGCCAGUUGAAGAACUGU	68	10,244	7212	746
	mir-26	12:hsa;mir-26a-2,57824622,57824643,-	mir-26a-2	77	20,298	14,246	1478	miR-26a-5p	5p-UUCAAGUAAUCCAGGAUAGGCU	76	20,295	14,289	1478
	mir-221	X:hsa;let-7f-2,53557199,53557220,-	let-7f-2	64	18,956	13,304	1380	let-7f-5p	5p-UGAGGUAGUAGAUUGUAUAGUU	64	18,956	13,346	1380
	let-7	3:hsa;let-7g,52268282,52268303,-	let-7g	56	15,713	11,028	1144	let-7g-5p	5p-UGAGGUAGUAGUUUGUACAGUU	54	15,709	11,060	1144
	mir-28	8:hsa;mir-151a,140732610,140732630,-	mir-151a	139	13,978	9810	1018	miR-151a-3p	3p-CUAGACUGAAGCUCCUUGAGG	109	10,663	7507	776
	mir-127	14:hsa;mir-127,100883035,100883056,+	mir-127	135	13,017	9136	948	miR-127-3p	3p-UCGGAUCCGUCUGAGCUUGGCU	126	12,990	9146	946
	let-7	9:hsa;let-7d,94178841,94178862,+	let-7d	75	11,189	7853	815	let-7d-5p	5p-AGAGGUAGUAGGUUGCAUAGUU	51	9603	6761	699
	mir-10	X:hsa;mir-221,45746221,45746243,-	mir-221	75	10,284	7218	749	miR-221-3p	3p-AGCUACAUUGUCUGCUGGGUUUC	47	21,761	15,321	1584
	mir-103	20:hsa;mir-103a-2,3917541,3917563,+	mir-103a-2	88	9053	6354	659	miR-103a-3p	3p-AGCAGCAUUGUACAGGGCUAUGA	86	9049	6371	659
	let-7	3:hsa;mir-28,188688834,188688855,+	mir-28	82	6351	4457	462	miR-28-3p	3p-AAGGAGCUCACAGUCUAUUGAG	73	6095	4291	444
**PC-7 (SRX716598)**				3580	1,291,292	906,254	94016			3121	1,250,049	880,088	91,013
	mir-486	8:hsa;mir-486-2,41660444,41660465,+	mir-486-2	1130	1,350,603	375,231	55,530	miR-486-5p	5p-UCCUGUACUGAGCCGCCCCGAG	1071	1,349,469	376,279	55,483
	mir-191	3:hsa;mir-191,49020633,49020655,-	mir-191	277	267,823	74,408	11,012	miR-191-5p	5p-CAACGGAAUCCCAAAAGCAGCUG	266	267,756	746,60	11,009
	let-7	22:hsa;let-7b,46113691,46113712,+	let-7b	481	257,858	71,639	10,602	let-7b-5p	5p-UGAGGUAGUAGGUUGUGUGGUU	439	257,625	71,835	10,592
	mir-25	13:hsa;mir-92a-1,91351361,91351382,+	mir-92a-1	589	243,120	67,545	9996	miR-92a-3p	3p-UAUUGCACUUGUCCCGGCCUGU	574	243,075	67,778	9994
	let-7	12:hsa;let-7i,62603691,62603712,+	let-7i	222	194,147	53,939	7982	let-7i-5p	5p-UGAGGUAGUAGUUUGUGCUGUU	214	194,099	54,122	7980
	let-7	9:hsa;let-7a-1,94175962,94175983,+	let-7a-1	206	182,459	50,692	7502	let-7a-5p	5p-UGAGGUAGUAGGUUGUAUAGUU	197	182,420	50,865	7500
	mir-181	1:hsa;mir-181a-1,198859067,198859089,-	mir-181a-1	253	108,155	30,048	4447	miR-181a-5p	5p-AACAUUCAACGCUGUCGGUGAGU	244	107,985	30,110	4440
	mir-320	8:hsa;mir-320a,22245007,22245028,-	mir-320a	813	106,945	29,712	4397	miR-320a-3p	3p-AAAAGCUGGGUUGAGAGGGCGA	780	106,785	29,775	4390
	mir-423	17:hsa;mir-423,30117095,30117117,+	mir-423	492	90,138	25,043	3706	miR-423-5p	5p-UGAGGGGCAGAGAGCGAGACUUU	267	65,916	18,380	2710
	let-7	3:hsa;let-7g,52268282,52268303,-	let-7g	104	50,033	13,900	2057	let-7g-5p	5p-UGAGGUAGUAGUUUGUACAGUU	102	50,027	13,949	2057
	mir-22	17:hsa;mir-22,1713955,1713976,-	mir-22	81	45,147	12,543	1856	miR-22-3p	3p-AAGCUGCCAGUUGAAGAACUGU	78	45,115	12,580	1855
	mir-26	12:hsa;mir-26a-2,57824622,57824643,-	mir-26a-2	135	44,947	12,487	1848	miR-26a-5p	5p-UUCAAGUAAUCCAGGAUAGGCU	134	44,945	12,532	1848
	mir-127	14:hsa;mir-127,100883035,100883056,+	mir-127	299	40,887	11,359	1681	miR-127-3p	3p-UCGGAUCCGUCUGAGCUUGGCU	276	40,818	11,381	1678
	mir-28	8:hsa;mir-151a,140732610,140732630,-	mir-151a	279	46,226	12,843	1901	miR-151a-3p	3p-CUAGACUGAAGCUCCUUGAGG	221	36,268	10,113	1491
	mir-221	X:hsa;let-7f-2,53557199,53557220,-	let-7f-2	96	31,868	8854	1310	let-7f-5p	5p-UGAGGUAGUAGAUUGUAUAGUU	95	31,865	8885	1310
	mir-10	X:hsa;mir-221,45746221,45746243,-	mir-221	178	27,407	7614	1127	miR-221-3p	3p-AGCUACAUUGUCUGCUGGGUUUC	169	27,340	7623	1124
	let-7	9:hsa;let-7d,94178841,94178862,+	let-7d	159	35,482	9858	1459	let-7d-5p	5p-AGAGGUAGUAGGUUGCAUAGUU	104	26,539	7400	1091
	mir-423	17:hsa;mir-423,30117131,30117153,+	mir-423	492	90,138	25,043	3706	miR-423-3p	3p-AGCUCGGUCUGAGGCCCCUCAGU	221	24,211	6751	995
	let-7	3:hsa;mir-28,188688834,188688855,+	mir-28	138	18,683	5191	768	miR-28-3p	3p-AAGGAGCUCACAGUCUAUUGAG	123	18,324	5109	753
	mir-103	20:hsa;mir-103a-2,3917541,3917563,+	mir-103a-2	163	16,403	4557	674	miR-103a-3p	3p-AGCAGCAUUGUACAGGGCUAUGA	159	16,386	4569	674
				6587	3,248,469	902,504	13,3561			5734	3,136,968	874,696	128,977

Items in the main columns of the table: gene family, coordinate string, pre-microRNA. UR, number of unique reads; RC, read count; RPM (lib), the read per million normalized by the total number of reads mapped to the library to a known microRNA; RPM (total), the reads per million normalized by the total number of genome mapped reads (genome mode) or the total number of reads in the analysis (sequence library mode); mature microRNAs sequence, miR 5p-/3p-arms, arm sequence as defined by the miRBase annotation. The order of classification of miRNAs is based on the RC.

**Table 3:** Quantification and detection of isomiR variants on platelets concentrates.

(1) Quantification of IsomiR on Platelets Concentrates									
Samples	NTA *n* (%)				length Variants *n* (%)				
	NTA(A)	NTA(U)	NTA(C)	NTA(G)	lv3pE	lv3pT	lv5pE	lv5pT	mv
PC-1: SRX716593	1,502,312 (37.44%)	2,447,590 (60.99%)	40,885 (1.02%)	22,103 (0.55%)	327,889 (23.06%)	1,003,147 (70.57%)	12,792 (0.90%)	40,465 (2.85%)	37,294 (2.62%)
PC-2: SRX716594	729,738 (32.24%)	1,491,187 (65.88%)	29,352 (1.30%)	13,064 (0.58%)	273,874 (26.53%)	687,827 (66.62%)	11,494 (1.11%)	29,902 (2.90%)	29,309 (2.84%)
PC-3: SRX716595	810,533 (32.93%)	1,608,776 (65.37%)	28,059 (1.14%)	13,731 (0.56%)	228,445 (25.58%)	599,868 (67.17%)	10,425 (1.17%)	27,552 (3.09%)	26,805 (3.0%)
PC-4: SRX716596	408,066 (30.47%)	891,951 (66.59%)	24,271 (1.81%)	15,139 (1.13%)	252,015 (32.18%)	485,983 (62.06%)	8226 (1.05%)	13,027 (1.66%)	23,772 (3.04%)
PC-5: SRX716597	248,172 (31.43%)	524,128 (66.38%)	11,835 (1.50%)	5399 (0.68%)	117,710 (28.07%)	276,958 (66.05%)	4360 (1.04%)	8447 (2.01%)	11,825 (2.82%)
PC-7: SRX716598	563,001 (30.42%)	1,245,683 (67.31%)	28,203 (1.52%)	13,850 (0.75%)	315,489 (30.11%)	666,785 (63.64%)	10,999 (1.05%)	23,665 (2.26%)	30,845 (2.94%)
(2) IsomiR quantification of miRNAs									
MicroRNAs	NTA(A)	NTA(U)	NTA(C)	NTA(G)	lv3pE	lv3pT	lv5pE	lv5pT	mv
	Mean ± SD	Mean ± SD	Mean ± SD	Mean ± SD	Mean ± SD	Mean ± SD	Mean ± SD	Mean ± SD	Mean ± SD
miR-486-5p	309,714.33 ± 214,491.01	574,218.67 ± 322,270.07	5408.50 ± 2232.18	3203.00 ± 1596.31	8734.00 ± 6386.92	164,491.33 ± 70,125.67	0.00 ± 0.00	1229.17 ± 695.15	488.50 ± 291.12
miR-92a-3p	24,452.83 ± 11,342.48	53,794.17 ± 24,607.88	1299.67 ± 567.11	441.00 ± 198.83	15,592.33 ± 5685.46	2098.67 ± 1071.13	0.00 ± 0.00	4383.33 ± 2515.53	1046.33 ± 581.30
miR-320a-3p	70.00 ± 26.11	36,580.33 ± 11,751.30	3276.83 ± 1017.55	586.50 ± 196.71	11,801.83 ± 4316.82	4569.67 ± 1424.88	316.33 ± 109.64	601.67 ± 222.68	1153.83 ± 405.98
miR-127-3p	24,75.50 ± 1398.62	23,384.33 ± 14,379.70	528.67 ± 284.26	135.17 ± 68.68	69.67 ± 39.70	2250.83 ± 1379.51	1.00 ± 1.67	23.67 ± 12.20	51.33 ± 30.78
let-7i-5p	5077.67 ± 1715.29	15,656.33 ± 5569.34	244.67 ± 94.76	63.33 ± 27.44	271.33 ± 91.98	13,603.17 ± 6109.05	49.17 ± 30.21	73.00 ± 42.30	3.17 ± 5.45
let-7b-5p	4275.00 ± 2339.25	15,041.50 ± 8794.91	276.67 ± 107.23	1220.33 ± 447.08	42,038.67 ± 23925.81	13,505.17 ± 5057.22	0.33 ± 0.81	27.17 ± 19.67	89.00 ± 74.70
miR-103a-3p	19.00 ± 10.25	6990.67 ± 5086.50	427.00 ± 299.86	83.50 ± 62.05	399.83 ± 304.29	3527.33 ± 2085.80	0.00 ± 0.00	107.33 ± 60.68	16.50 ± 14.39
miR-151a-3p	1053.67 ± 520.25	5477.33 ± 2625.91	1695.17 ± 745.09	12.67 ± 8.35	12,337.17 ± 5300.43	194.83 ± 128.54	1263.83 ± 479.98	18.17 ± 15.14	489.50 ± 191.70
miR-221-3p	255.83 ± 92.34	5208.17 ± 2445.48	39.83 ± 18.62	98.50 ± 43.19	233.67 ± 103.63	8629.50 ± 3483.21	0.83 ± 1.32	31.83 ± 21.29	18.33 ± 11.37
miR-26a-5p	326.67 ± 140.92	2566.17 ± 947.53	202.00 ± 83.77	99.33 ± 64.75	98.00 ± 43.78	814.00 ± 251.09	0.00 ± 0.00	90.00 ± 31.56	3.00 ± 2.44
miR-423-3p	629.83 ± 262.84	2468.17 ± 523.97	42.33 ± 17.09	21.33 ± 6.71	34.50 ± 10.15	1111.50 ± 505.16	1174.50 ± 270.23	91.83 ± 30.45	244.00 ± 96.06
miR-28-3p	3834.83 ± 1724.38	2080.50 ± 861.14	1169.50 ± 506.45	9.83 ± 7.19	313.67 ± 176.53	451.17 ± 194.87	8.83 ± 3.86	88.33 ± 33.26	641.33 ± 309.20
miR-423-5p	1027.50 ± 347.28	1931.33 ± 692.43	246.67 ± 79.94	73.17 ± 28.13	8093.67 ± 2410.61	10,057.67 ± 3071.29	3.50 ± 3.72	56.00 ± 21.75	106.83 ± 42.37
miR-181a-5p	1010.00 ± 364.20	995.00 ± 335.88	64.17 ± 36.45	496.00 ± 211.93	4112.50 ± 2167.39	59,343.00 ± 22,151.37	24.17 ± 9.04	32.17 ± 13.25	147.00 ± 46.51
miR-191-5p	593.00 ± 377.16	354.17 ± 359.66	138.00 ± 61.92	394.33 ± 220.96	18,876.83 ± 8085.89	3657.83 ± 3352.16	0.00 ± 0.00	6858.50 ± 4181.14	688.33 ± 335.25
let-7g-5p	560.33 ± 291.29	102.83 ± 35.91	24.00 ± 13.08	87.17 ± 33.34	1824.00 ± 654.24	3554.83 ± 1964.37	193.67 ± 74.25	4.50 ± 5.35	33.67 ± 16.21
let-7a-5p	1487.17 ± 713.56	34.17 ± 15.19	112.67 ± 37.51	250.83 ± 85.09	13,054.83 ± 4155.43	10,072.00 ± 3754.47	3.83 ± 3.65	54.33 ± 51.65	44.17 ± 18.68
let-7d-5p	251.00 ± 93.41	9.33 ± 6.71	15.83 ± 8.97	45.50 ± 16.02	2268.83 ± 731.15	1409.00 ± 563.21	10.00 ± 4.00	74.83 ± 40.87	18.33 ± 10.23
miR-22-3p	63.00 ± 24.42	6.83 ± 6.17	5.17 ± 1.94	54.83 ± 20.99	60.83 ± 39.65	1100.67 ± 466.98	3.17 ± 2.85	79.83 ± 42.65	1.67 ± 1.50
let-7f-5p	343.50 ± 128.08	1.33 ± 2.42	25.33 ± 9.58	3.50 ± 3.20	1374.67 ± 384.31	2608.00 ± 1280.50	14.00 ± 7.07	16.00 ± 15.33	7.83 ± 5.34

IsomiR types: NTA (non-templated additions), A (adenine addition), C (cytosine addition); U (U/T addition, (U) uracil and (T) thymine); G (guanine addition). NTA(A), number of reads with a non-templated A addition; NTA(U), number of reads with a non-templated U addition; NTA(C), number of reads with a non-templated C addition; NTA(G), number of reads with a non-templated G addition. Length variants: lv3pE, number of reads with 3′ length extension (longer than the canonical sequence); lv3pT, number of reads with 3′ length trimming (shorter than the canonical sequence); lv5pE, number of reads with 5′ length extension (longer than the canonical sequence); lv5pT, number of reads with 5′ length extension (shorter than the canonical sequence); mv, number of reads classified as multiple length variants. All isomiRs are quantified in read count. ANOVA was used to test the means in each group of isomiRs with *p*-value <0.001 in all groups tested. *n* (%), number and the percentage value of isomiRs, respectively; SD, standard deviation.

## Supplementary Materials

The following are available online https://www.mdpi.com/2218-273X/10/8/1173/s1, File S1: miRNAs-canonicos.xlsx, File S2: Mature.iso.xlsx, File S3: Enrichment analysis.xlsx.
